# Extended-Spectrum Beta-Lactamase and Carbapenem-Resistant Gram-Negative Pathogens in Makkah, Saudi Arabia

**DOI:** 10.4314/ejhs.v32i6.20

**Published:** 2022-11

**Authors:** Ahmed Kabrah

**Affiliations:** 1 Laboratory Medicine Department, Faculty of Applied Medical Sciences, Umm Al-Qura University, Makkah, Kingdom of Saudi Arabia

**Keywords:** Carbapenem, Extended-spectrum beta-lactamase, Gram-negative bacteria, Resistant, Saudi Arabia

## Abstract

**Background:**

This study aimed to determine the prevalence of extended-spectrum-beta-lactamase (ESBL) and carbapenem-resistant gram-negative bacteria (GNB) isolated from patients at the King Faisal Hospital in Makkah, Saudi Arabia.

**Methods:**

In this cross-sectional study, a total of 298 patients admitted to the intensive care unit for 48 hours and who had a central venous catheter were selected using a census sampling method. Only patients with ESBL and carbapenem-resistant GNB-isolated organisms (175 patients) were included. The susceptibility test of GNB was carried out according to the standard recommendations. The identified strains were tested in-vitro against several antimicrobial drugs. Statistical analysis was performed using SPSS version 24.

**Results:**

36(20.6%) of samples were ESBL-producing GNB, whereas 139(79.4%) were carbapenem-resistant GNB. The pooled proportional estimates of ESBL-producing GNB Escherichia coli, Klebsiella pneumoniae, and other GNB were 44.4%, 41.6%, and 14.0%, respectively; the pooled proportional estimates of carbapenem resistance GNB Klebsiella pneumoniae, Acinetobacter baumannii complex/hemolyticus and other GNB were 82.8%, 10.8%, and 6.4%, respectively. All ESBL-producing GNB and carbapenem-resistance GNB were multidrug-resistant pathogens. The highest carbapenem resistance GNB 139(100%) was to ampicillin, and the lowest 122(87.7%) was to Amoxicillin/clavulanic acid (Amox/clav). All ESBL-producing GNB 36 (100%) were resistant to cefotaxime, and 35 (97.2%) were resistant to ampicillin, cefuroxime, cefepime, and ceftazidime. Additionally, the effective antibiotic against ESBL-producing GNB was imipenem.

**Conclusion:**

Antibiotic utilization measures appear to contribute to the control of the emergence of multidrug-resistant pathogens such as ESBL and carbapenem-resistant GNB. Strict adherence to well-accepted infection control guidelines along with caution in using broad-spectrum antimicrobial agents represents the best strategy for preventing the emergence and spread of multidrug-resistant pathogens.

## Introduction

Antimicrobial resistance (AMR) is a global health problem associated with high morbidity and mortality rates (1). The load of AMR is growing, with transmission often happening in healthcare settings due to poor infection control practices and improper use of antibiotics (2). Several studies have highlighted that it is the best time to take the necessary initiatives to prevent the emergence and spread of AMR pathogens (3–5).

AMR strains have been found in Saudi Arabia, and currently, several studies are being conducted to have a more in-depth view (6–8). Gram-negative bacteria (GNB) and *Enterobacteriaceae* are one of the most common pathogenic organisms causing various life-threatening severe infections (9). Notably, carbapenems are the most effective antibiotic therapy for infections caused by multidrug-resistant *Enterobacteriaceae* (10, 11). With the widespread use of carbapenem antibiotics, carbapenem-resistant *Enterobacteriaceae* have extensively developed and become a serious public health concern (12). The emergence of extended-spectrum beta-lactamase (ESBL) and carbapenem resistance bacterial infections among intensive care unit (ICU) patients is one of the most alarming global health threats today. They are significant reasons for morbidity, more extended hospital stays, and mortality in the ICU with limited therapeutic options, and they utilize many sources to prevent, identify, and respond to them (13). Therefore, GNB isolates from urinary tract infections, bloodstream infections, lower respiratory tract infections, and wound infections in ICUs are superbugs and nightmare bacteria (14).

ESBLs are enzymes produced by certain bacteria that can hydrolyze extended-spectrum third-generation cephalosporins (15). In contrast, carbapenemase is a class of beta-lactamase enzymes released from GNB to protect itself against carbapenem antibiotics and leads to giving resistance to all penicillins and cephalosporins (16). In Saudi Arabia, the GNB shows a meaningful increase in becoming carbapenem-resistant GNB, which is significantly higher than in other parts of the globe (17). In addition, an increasing predominance of ESBL has been found in Saudi Arabia. It has been further defined that *Klebsiella pneumoniae* shows 65% ESBL, and *Escherichia coli* gives 29% ESBL rates. It is causing an expansion in Saudi Arabia's mortality rate from 11% to 40% through multiple reported mortality outbreaks of uncontrollable infections due to AMR pathogen infections (18). Furthermore, the rate of carbapenem-resistant *Acinetobacter baumannii* has overgrown in the last five years in Saudi Arabia (19). Additionally, according to the current surveillance conducted on Gram-positive cocci in Saudi Arabia, 32% of *Staphylococcus aureus* have become methicillinresistant, 33% of *Streptococcus pneumoniae* have become resistant to penicillin G, and another 26% have become utterly resistant to erythromycin (20). Therefore, this study aimed to determine the prevalence of ESBL and carbapenem-resistant GNB isolated from patients at the King Faisal Hospital in Makkah, Saudi Arabia.

## Methods

**Study design, setting, and period**: This cross-sectional study was conducted at the King Faisal Hospital in Makkah, Saudi Arabia, between November 1, 2020, and January 31, 2021.

**Data collection procedure**: Samples were collected from patients admitted to the ICU for 48 hours and who had a central venous catheter (CVC). Samples were collected from different locations as follows: blood, central vein puncture, sputum, urine culture, wound, and bronchoalveolar lavage.

Blood drawing procedures were done by hospital nursing, which follows the Ministry of Health regulation that includes cleaning the skin with 2% chlorhexidine with a 70% isopropyl alcohol applicator for 30 seconds using a back-and-forth scrubbing technique. Besides, two sets of blood cultures were drawn from patients to rule out the contamination. The samples were sent to a reference lab for molecular identification.

**Sample size and sampling**: The study participants were selected using the census sampling method as all eligible patients were found during the study period (between November 1, 2020, and January 31, 2021); 298 patients who were admitted to the ICU for 48 hours and who had a CVC were eligible to be included in the study. Only patients with ESBL and carbapenem-resistant GNB-isolated organisms (175 patients) were included in this study. Outpatients and patients with other types of microorganisms were excluded from the study. One sample was obtained from each patient according to his/her medical condition.

Isolation and identification of pathogens

The King Faisal Hospital Microbiology Laboratory proceeded with the received samples from the ICU according to the standard operating procedures for bacterial isolation and identification. Blood culture bottles were incubated in Biomeieux BACT/ALERT. After the machine gave the growth signal, the samples were cultured in blood, MacConkey, and chocolate agar. Urine samples were cultured in blood, MacConkey, or CLED agar. Lower respiratory tract samples were cultured in blood, MacConkey, and chocolate agar. They have then incubated aerobically at 37°C for 24 to 48 hours, except for the blood culture, which was incubated for 5 to 7 days. Preliminary identification of some isolates was performed based on the colonial morphology, Gram stain, and routine rapid biochemical tests such as catalase, indole, and oxidase tests. Bacterial identification was performed according to the protocols of the hospital where the strain originated: gram-negative strains were identified by Pos Breakpoint Combo Panel Type 50 in MicroScan (Beckman Coulter Inc., CA, United States), and gram-positive strains by Pos Breakpoint Combo Panel Type 28 in MicroScan (Beckman Coulter Inc., CA, United States).

**Antimicrobial susceptibility testing**: The susceptibility test of GNB was carried out according to the recommendations of the Clinical and Laboratory Standards Institute (CLSI). Identified strains were tested in vitro against several classes of antimicrobial drugs using the MicroScan automated microbiology system (Pos Breakpoint Combo 50 Panel). The following antimicrobial agents were examined: Amox/clav (2 µg/ml), ampicillin (16 µg/ml), cefepime (16 µg/ml), cefotaxime (16 µg/ml), ceftazidime (16 µg/ml), ciprofloxacin (2 µg/ml), cefuroxime (16 µg/ml), gentamicin (8 µg/ml), imipenem (8 µg/ml), meropenem (8 µg/ml), and trimeth/Sulfa (0.05 µg/ml). Quality control and maintenance were achieved according to the manufacturer's guidelines.

**Ethical considerations**: The study protocol was approved by the Research and Ethical Committee of the College of Medicine at Umm Al Qura University (HAPO-02-K-012-2021-08-713). Also, permission was obtained from the King Faisal Hospital. The conscious patients and unconscious patients' proxies who agreed to participate in the study were asked to sign a written informed consent for participation in the study.

**Statistical analysis**: The Statistical Package for Social Science (SPSS) version 24 (IBM Corp, Armonk, NY, USA) was used for data analysis, including descriptive statistics of frequencies and percentages.

## Results

A total of 175 ESBL and carbapenem-resistant GNB-isolated organisms' samples were collected from blood n = 28 (16.0%), central vein puncture n = 7 (4.0%), sputum n = 71 (40.6%), urine culture n = 35 (20.0%), wound n = 33 (18.9%), and bronchoalveolar lavage n = 1 (0.5%) of patients admitted to the ICU for 48 hours and had a CVC in the King Faisal Hospital between November 1, 2020 and January 31, 2021. 120 (65.6%) samples were from males, and 55 (31.4%) samples were from female patients. 36 (20.6%) samples were positive for ESBL-producing GNB, of which 23.0 (63.9%) samples were from males, and 13.0 (36.1%) samples were from females. 139 (79.4%) samples were positive for carbapenem resistance in GNB, of which 97.0 (69.8%) samples were from males, and 42.0 (30.2%) samples were from females (Table 1).

**Table 1:** The sources of isolated microorganisms for the ESBL-producing GNB and carbapenem-resistant GNB by gender

Sample sources	ESBL-producing GNB n = 36 (%)		Carbapenem resistance GNB n = 139 (%)		Total n=175 (%)

	Male	Female	Male	Female	
Blood	2 (8.7)	1 (7.7)	14 (14.4)	11 (26.2)	28 (16.0)
Central vein puncture	0 (0.0)	1 (7.7)	6.0 (6.2)	0 (0.0)	7 (4.0)
Sputum	9 (39.1)	1 (7.7)	43 (44.3)	18 (42.9)	71 (40.6)
Urine culture	5 (21.8)	8 (61.5)	16 (16.5)	6 (14.2)	35 (20.0)
Wound	7 (30.4)	2 (15.4)	17 (17.5)	7 (16.7)	33 (18.9)
Broncho alveolar lavage	0 (0.0)	0 (0.0)	1 (1.1)	0 (0.0)	1 (0.5)
Total	23 (63.9)	13 (36.1)	97 (69.8)	42 (30.2)	175 (100.0)

ESBL: Extended-Spectrum Beta-Lactamase; GNB: Gram-negative bacteria

Table 2 shows the types of ESBL-producing GNB-isolated organisms by the sources of the samples. 3.0 (8.3%) blood samples were positive for ESBL-producing GNB, of which 1 (33.3%) sample was *Escherichia coli*, and 2.0 (66.7%) samples were *Klebsiella pneumoniae*. 1 (100.0) central vein puncture sample was ESBL-producing GNB-isolated *Klebsiella pneumoniae*. 10 (27.8%) sputum samples were positive for ESBL-producing GNB, of which 1 (10.0%) sample was *Enterobacter aerogenes*, 1 (10.0%) sample was *Enterobacter cloacae*, 2 (20.0%) samples were *Escherichia coli*, 1 (10.0%) sample was *Klebsiella oxytoca*, and 5 (50.0%) samples were *Klebsiella pneumoniae*. 13 (36.1%) urine culture samples were positive for ESBL-producing GNB, of which 1 (7.7%) sample was *Citrobacter freundii*, 9 (69.2%) samples were *Escherichia coli*, and 3 (23.1%) samples were *Klebsiella pneumoniae*. 24 (17.3%) wound samples were positive for ESBL-producing GNB, of which 1 (11.2%) sample was *Enterobacter cloacae*, 4 (44.4%) samples were *Escherichia coli*, and 4 (44.4%) samples were *Klebsiella pneumoniae*. All bronchoalveolar lavage samples were negative for ESBL-producing GNB. In addition, the results demonstrated that the pooled proportional estimates of ESBL-producing GNB-isolated organisms.

**Table 2:** Types of ESBL-producing GNB-isolated organisms by the sources of the samples

Isolated organisms	Blood	Central vein puncture	Sputum	Urine culture	Wound	Broncho alveolar lavage	Total n = 36 (%)
*Citrobacter freundii*	0 (0.0)	0 (0.0)	0 (0.0)	1 (7.7)	0 (0.0)	0 (0.0)	1 (2.8)
*Enterobacter aerogenes*	0 (0.0)	0 (0.0)	1 (10.0)	0 (0.0)	0 (0.0)	0 (0.0)	1 (2.8)
*Enterobacter cloacae*	0(0.0)	0 (0.0)	1 (10.0)	0 (0.0)	1 (11.2)	0 (0.0)	2 (5.6)
*Escherichia coli*	1 (33.3)	0 (0.0)	2 (20.0)	9 (69.2)	4(44.4)	0 (0.0)	16 (44.4)
*Klebsiella oxytoca*	0 (0.0)	0 (0.0)	1 (10.0)	0 (0.0)	0 (0.0)	0 (0.0)	1 (2.8)
*Klebsiella pneumoniae*	2 (66.7)	1(100.0)	5 (50.0)	3 (23.1)	4 (44.4)	0 (0.0)	15 (41.6)
Total	3 (8.3)	1 (2.8)	10 (27.8)	13 (36.1)	9 (25)	0 (0.0)	36 (100)

Extended-Spectrum Beta-Lactamase; GNB: Gram-negative bacteria

*Escherichia coli, Klebsiella pneumoniae, Enterobacter cloacae*, and other GNB were 44.4%, 41.6%, 5.6%, and 8.4%, respectively.

Table 3 shows the types of carbapenem resistance in GNB isolated organisms by the sources of the samples. 25 (18.0%) blood samples were positive for carbapenem resistance in GNB isolated organisms, of which 20 (80.0%) samples were *Klebsiella pneumoniae.* 6 (4.3%) central vein puncture samples were positive for carbapenem resistance in GNB isolated organisms, of which 4 (66.6%) samples were *Klebsiella pneumoniae.* 61 (43.9%) sputum samples were positive for carbapenem resistance in GNB isolated organisms, of which 49 (80.3%) samples were *Klebsiella pneumoniae.* 22 (15.8%) urine culture samples were positive for carbapenem resistance in GNB isolated organisms, of which 19 (86.5%) samples were *Klebsiella pneumoniae.* 24 (17.3%) wound samples were positive for carbapenem resistance in GNB isolated organisms, of which 22 (91.6%) samples were *Klebsiella pneumoniae*. Only 1 (0.7%) wound sample was positive for carbapenem resistance in GNB isolated *Klebsiella pneumoniae.* Moreover, the results demonstrated the pooled proportional estimates of carbapenem resistance in GNB isolated organisms *Klebsiella pneumoniae, Acinetobacter baumannii complex/hemolyticus, Pseudomonas aeruginosa*, and other GNB were 82.8%, 10.8%, 2.2%, and 4.2%, respectively.

**Table 3:** Types of carbapenem resistance in GNB isolated organisms by the sources of the GNB: Gram-negative bacteria

Isolated organisms	Blood	Central vein puncture	Sputum	Urine culture	Wound	Broncho alveolar lavage	Total n = 139 (%)
*Acinetobacter baumannii* *complex/hemolyticus*	4 (16.0)	1 (16.7)	8 (13.1)	1 (4.5)	1 (4.2)	0 (0.0)	15 (10.8)
*Enterobacter aerogenes*	0 (0.0)	0 (0.0)	0 (0.0)	1 (4.5)	0 (0.0)	0 (0.0)	1 (0.7)
*Escherichia coli*	0 (0.0)	0 (0.0)	0 (0.0)	1 (4.5)	0 (0.0)	0 (0.0)	1 (0.7)
*Klebsiella oxytoca*	0 (0.0)	0 (0.0)	2 (3.3)	0 (0.0)	0 (0.0)	0 (0.0)	2 (1.4)
*Klebsiella pneumoniae*	20 (80.0)	4 (66.6)	49(80.3)	19 (86.5)	22 (91.6)	1 (100)	115 (82.8)
*Morganella morganii*	0 (0.0)	1 (16.7)	0 (0.0)	0 (0.0)	0 (0.0)	0 (0.0)	1 (0.7)
*Proteus mirabilis*	1 (4.0)	0 (0.0)	0 (0.0)	0 (0.0)	0 (0.0)	0 (0.0)	1 (0.7)
*Pseudomonas* *aeruginosa*	0 (0.0)	0 (0.0)	2 (3.3)	0 (0.0)	1 (4.2)	0 (0.0)	3 (2.2)
Total	25 (18.0)	6 (4.3)	61 (43.9)	22 (15.8)	24 (17.3)	1 (0.7)	139 (100)

Table 4 presents the ESBL-producing GNB resistance to the examined ten antibiotics. The results demonstrate that all ESBL-producing GNBs such as *Citrobacter freundii, Enterobacter aerogenes, Enterobacter cloacae, Escherichia coli, Klebsiella oxytoca, and Klebsiella pneumoniae* were multidrug-resistant bacteria. 36 (100%) ESBL-producing GNB were resistant to cefotaxime, and 35 (97.2%) were resistant to ampicillin, cefuroxime, cefepime, and ceftazidime. On the contrary, Among the examined ten antibiotics (Ampicillin, cefuroxime, cefepime, ceftazidime, imipenem, cefotaxime, gentamicin, ciprofloxacin, amox/clav, and trimeth/sulfa), the effective antibiotic against ESBL-producing GNB was imipenem.

**Table 4:** ESBL-producing GNB resistance to the most commonly used antibiotics

Isolated organism	Ampicillin	Cefuroxime	Cefepime	Ceftazidime	Imipenem	Cefotaxime	Gentamicin	Ciprofloxacin	Amox/clav	Trimeth/ Sulfa
*Citrobacter freundii* (n=1)	1 (100)	1 (100)	1 (100)	1 (100)	0 (0.0)	1 (100)	1 (100)	1 (100)	0 (0.0)	1 (100)
*Enterobacter aerogenes* (n=1)	1 (100)	1 (100)	1 (100)	1 (100)	0 (0.0)	1 (100)	0 (0.0)	1 (100)	1 (100)	1 (100)
*Enterobacter cloacae* (n=2)	2 (100)	2 (100)	2 (100)	2 (100)	0 (0.0)	2 (100)	1 (50)	1 (50)	1 (50)	1 (50)
*Escherichia coli* (n=16)	16 (100)	16 (100)	16 (100)	16 (100)	0 (0.0)	16 (100)	4 (25.0)	9 (56.2)	3 (18.7)	9 (56.2)
*Klebsiella oxytoca* (n=1)	1 (100)	1 (100)	1 (100)	1 (100)	0 (0.0)	1 (100)	1 (100)	1 (100)	1 (100)	1 (100)
*Klebsiella pneumoniae* (n=15)	14 (93.3)	14 (93.3)	14 (93.3)	14 (93.3)	1 (6.6)	14 (93.3)	7 (46.6)	8 (53.3)	3 (20.0)	14 (93.3)
Total	35 (97.2)	35 (97.2)	35 (97.2)	35 (97.2)	1 (2.77)	36 (100)	15 (41.6)	22 (61.1)	9 (25.0)	28 (77.7)

ESBL: Extended-Spectrum Beta-Lactamase; GNB: Gram-negative bacteria

Table 5 presents the distribution of carbapenem resistance GNB to the examined ten antibiotics. The results demonstrate that all carbapenem resistance GNB as *Acinetobacter baumannii complex/ hemolyticus, Enterobacter aerogenes, Escherichia coli, Klebsiella oxytoca, Klebsiella pneumoniae, Morganella morganii, Proteus mirabilis, and Pseudomonas aeruginosa* were multidrug-resistant bacteria. The highest carbapenem resistance GNB 139 (100%) were resistant to ampicillin. In contrast, the lowest carbapenem resistance GNB 122 (87.7%) was resistant to amox/clav.

**Table 5:** Distribution of carbapenem resistance GNB by the most commonly used antibiotics

GNB isolates	Ampicillin	Cefuroxime	Meropenem	Ceftazidime	Imipenem	Cefotaxime	Gentamicin	Ciprofloxacin	Amox/clav	Trimeth/ Sulfa
*Acinetobacter baumannii* *complex/ hemolyticus* (n=15)	15 (100)	15 (100)	15 (100)	15 (100)	15 (100)	15 (100)	15 (100)	14 (93.3)	1 (6.6)	15 (100)
*Enterobacter aerogenes* (n=1)	1 (100)	1 (100)	1 (100)	1 (100)	1 (100)	1 (100)	1 (100)	1 (100)	1 (100)	1 (100)
*Escherichia coli* (n=1)	1 (100)	1 (100)	0 (0.0)	1 (100)	0 (0.0)	0 (0.0)	0 (0.0)	0 (0.0)	1 (100)	0 (0.0)
*Klebsiella oxytoca* (n=2)	2 (100)	2 (100)	2 (100)	2 (100)	2 (100)	2 (100)	2 (100)	2 (100)	2 (100)	2 (100)
*Klebsiella pneumoniae* (n=115)	115 (100)	115 (100)	115 (100)	115 (100)	115 (100)	115 (100)	115 (100)	115 (100)	115 (100)	115 (100)
*Morganella morganii* (n=1)	1 (100)	1 (100)	0 (0.0)	1 (100)	0 (0.0)	0 (0.0)	0 (0.0)	1 (100)	1 (100)	1 (100)
*Proteus mirabilis* (n=1)	1 (100)	1 (100)	0 (0.0)	0 (0.0)	1 (100)	0 (0.0)	1 (100)	1 (100)	1 (100)	1 (100)
*Pseudomonas aeruginosa* (n=3)	3 (100)	3 (100)	3 (100)	3 (100)	3 (100)	0 (0.0)	3 (100)	3 (100)	0 (0.0)	0 (0.0)
Total	139 (100)	136 (97.8)	136 (97.8)	138 (99.2)	137 (98.5)	133 (95.6)	137 (98.5)	137 (98.5)	122 (87.7)	135 (97.1)

## Discussion

AMR is one of the biggest threats to global health, food security, and development today, which can affect anyone, of any age, in any country. In addition, AMR occurs naturally, but misuse of antibiotics in humans and animals is accelerating the process. Furthermore, AMR leads to longer hospital stays, higher medical costs, and increased mortality (21). This study aimed to determine the prevalence of ESBL and carbapenem-resistant GNB isolated from patients at the King Faisal Hospital in Makkah, Saudi Arabia. In the current study, a total of 175 GNB samples were collected from patients admitted to the ICU for 48 hours and who had a CVC. The GNB samples were collected from different locations as follows: blood n = 28 (16.0%), central vein puncture n = 7 (4.0%), sputum n = 71 (40.6%), urine culture n = 35 (20.0%), wound n = 33 (18.9%), and bronchoalveolar lavage n = 1 (0.5%). The main results of the current study demonstrate that 36 (20.6%) of samples were ESBL-producing GNB, whereas 139 (79.4%) were carbapenem resistance GNB. Alebel et al. 2021 showed that 24.8% and 5.2% of the patients in ICU have infections with ESBL and carbapenemase releasing GNB, respectively (13). Abayneh et al. 2020 meta-analysis showed that the overall proportional estimate of ESBL-producing GNB was 48.9% (22). Patients are often hospitalized in ICUs following problems in their respiratory, cardiac, renal, and brain after surgery and major trauma complications (23, 24). Patients hospitalized in ICUs are most at risk of developing infections from various sites (25). The most common infections that occur in the ICU are ventilator-associated lower respiratory tract infections, central line-associated bloodstream infections, and urinary tract infections associated with catheter and surgical site infections (26). Globally, infections with GNB are five to ten times higher among patients in the ICU than those in the general wards (27).

In addition, the results revealed that the pooled proportional estimates of ESBL-producing GNB Escherichia coli, Klebsiella pneumoniae, Enterobacter cloacae, and other GNB were 44.4%, 41.6%, 5.6%, and 8.4%, respectively. Abayneh et al. 2020 meta-analysis showed that the pooled proportional estimates of ESBL-producing Klebsiella pneumoniae, Escherichia coli, and other GNB were 61.8%, 41.2%, and 42.9%, respectively (22). Furthermore, Alebel et al. 2021 showed that the most frequent ESBL-producing isolates were Klebsiella pneumoniae (82.8%) and Escherichia coli (64%) (13). The results of the current study support these findings. Additionally, the results of the present study showed all ESBL-producing GNB were multidrug-resistant bacteria. Moreover, in the current study, all ESBL-producing GNB were resistant to cefotaxime, and 97.2% were resistant to ampicillin, cefuroxime, cefepime, and ceftazidime. On the contrary, the effective antibiotic against ESBL-producing GNB was imipenem. Multidrug-resistant is increasingly seen in many GNBs as a result of the widespread use of various antibiotics (28, 29).

ESBLs are enzymes that commonly mediate resistance to β-lactam antimicrobial drugs in GNB (30) and are most commonly found in Escherichia coli and Klebsiella pneumoniae (29). ESBLs have the ability to hydrolyze penicillins, third-generation cephalosporins, and monobactams (31). ESBL enzymes are encoded by transferable conjugative plasmids, which often code resistance determinants to other classes of antimicrobial agents and are also responsible for the dissemination of resistance to other GNBs in the community and in hospitals [32]. Infection caused by ESBL-producing bacteria is an emerging problem in the community setting, in hospitals, and in many parts of the world (33). Several reports have addressed the fecal carriage of these organisms during nosocomial outbreaks (31, 34). On the other hand, the results of the current study demonstrated the pooled proportional estimates of carbapenem resistance GNB Klebsiella pneumoniae, Acinetobacter baumannii complex/hemolyticus, Pseudomonas aeruginosa, and other GNB were 82.8%, 10.8%, 2.2%, and 4.2%, respectively. Enterobacterales family, Pseudomonas aeruginosa, and Acinetobacter species are potential superbugs that can produce ESBL, and carbapenemase-releasing GNB is widely reported in ICU settings. Moreover, the results of the present study showed all ESBL-producing GNB and carbapenem resistance GNB were multidrug-resistant bacteria. The prevailing prevalence of multidrug-resistant is in accord with studies done in Uganda (58%) (35), India (72.5%) (36), Nepal (62.1% and 79%) (37, 38), and Mexico (70.96%) (39). The high burden of multidrug-resistant isolates in the present study could be associated with empirical, non-selective use of antibiotics and irrational dose regimens. In the present study, the highest carbapenem resistance GNB of 100% was to ampicillin; and the lowest 87.7% was to amox/clav. Ampicillin is frequently used as the first line of treatment for common infections. Comparable results were reported in Ethiopia, where bacterial isolates were 100% resistant to ampicillin and 81.9% resistant to sulfamethoxazole-trimethoprim (40). Actually, the determinants of ESBL and carbapenem-resistant GNB among patients in the ICU need more studies in the future.

The main strength of our study was it is the first study that shows the prevalence of ESBL and carbapenem-resistant GNB isolated from patients admitted to the ICU for 48 hours and who had CVC at the King Faisal Hospital in Makkah, Saudi Arabia. The main limitation of this study is its cross-sectional design which limits the generalizability of our results. Furthermore, as the study was cross-sectional, there was no follow-up for the study participants. Additionally, all of the study isolates were collected from inpatients, and the exact number of nosocomial versus community-acquired bacteria was not differentiated.

In conclusion, our study demonstrates that 20.6% of samples were ESBL-producing GNB, whereas 79.4% were carbapenem resistance GNB. The pooled proportional estimates of ESBL-producing GNB Escherichia coli, Klebsiella pneumoniae, Enterobacter cloacae, and other GNB were 44.4%, 41.6%, 5.6%, and 8.4%, respectively. Whereas the pooled proportional estimates of carbapenem resistance GNB Klebsiella pneumoniae, Acinetobacter baumannii complex/hemolyticus, Pseudomonas aeruginosa, and other GNB were 82.8%, 10.8%, 2.2%, and 4.2%, respectively. All ESBL-producing GNB and carbapenem resistance GNB were multidrug-resistant bacteria. The highest carbapenem resistance GNB (100%) was to ampicillin, and the lowest (87.7%) was to amox/clav. All ESBL-producing GNB were resistant to cefotaxime, and 97.2% were resistant to ampicillin, cefuroxime, cefepime, and ceftazidime. On the contrary, the effective antibiotic against ESBL-producing GNB was imipenem. Therefore, antibiotic utilization measures appear to contribute to the control of the emergence of multidrug-resistant pathogens such as ESBL and carbapenem-resistant GNB. Strict adherence to well-accepted infection control guidelines along with caution in using broad-spectrum antimicrobial agents represents the best strategy for preventing the emergence and spread of nosocomial multidrug-resistant pathogens. Further future studies are required to confirm these findings.
